# “Make it the done thing”: an exploration of attitudes towards rest breaks, productivity and wellbeing while working from home

**DOI:** 10.1007/s00420-023-01985-6

**Published:** 2023-06-03

**Authors:** Lucy Walker, Elizabeth C. Braithwaite, Marc V. Jones, Steve Suckling, Danielle Burns

**Affiliations:** 1 Department of Psychology, Manchester Metropolitan University, Manchester, UK; 2 The Maslow Foundation, Ipswich, UK

**Keywords:** Rest breaks, Productivity, Wellbeing

## Abstract

**Objective:**

Taking regular rest breaks while working positively impacts productivity and wellbeing. While home and hybrid working styles have become a popular choice for employees, the impact of, and perceptions towards, taking breaks while working at home is poorly understood. The current research aimed to explore attitudes towards taking rest breaks while working from home and capture levels of breaks taken, wellbeing and productivity in a sample of UK white-collar workers.

**Methods:**

A mixed method approach was applied where self-report data from an online survey were gathered from individuals (*N* = 140) from one organisation. Open-ended questions regarding attitudes and perceptions towards rest break behaviours were obtained. Further quantitative measures included the number of breaks taken while working from home, levels of productivity (measured by the Health and performance Presenteeism subscale) and mental wellbeing (measured by the Short Warwick-Edinburgh Mental wellbeing scale). Both quantitative and qualitative analysis approaches were applied.

**Results:**

Qualitative responses indicated two overarching themes (1) Personal and (2) Organisational sat above four further themes including Movement outside, Structure of home working, Home environment and Digital presence. Additionally, quantitative findings indicated that the number of breaks taken outside was associated with positive changes in wellbeing.

**Conclusion:**

Employers could aim to support employees working from home in taking outside breaks through flexible working patterns, authentic leadership, and a change in company social norms around break behaviours. Such organisational changes could help to improve workforce productivity and wellbeing.

## Introduction

Homeworking, similar to remote working or teleworking, is defined as any paid work (minimum of 20 h per week) that is carried out primarily from home (Crosbie and Moore 2004). In 2019, approximately 1.7 million people reported working from home, representing 5% of the total UK workforce (ONS 2019). Since then, home working has accelerated, both because of technological advances (Felstead 2012) and the COVID-19 pandemic. During the first UK COVID-19 lockdown, almost half (45%) of working age adults reported working from home (Felstead and Reuschke 2020); a huge rise from pre-pandemic levels. Following the easing of the COVID-19 restrictions, home working and hybrid working (a mix of working from home and the work place) continues to be popular, with around 14% of workers exclusively working from home and 24% engaging in hybrid working (Office for National Statistics 2022). These working patterns are likely to continue, with more than half (54%) of employers in the information and communication industry stating that home working will be a key part of their business models moving forward (Office for National Statistics 2022).

There is evidence that homeworking is associated with increased productivity (Bloom et al. 2015; Dubrin 1991), improvements in work–life balance (Perry-Smith and Blum 2000) increased wellbeing linked to reduced pressure (Collins et al. 2016) and increased flexibility (Bosua et al. 2013). Conversely, home working may also be associated with negative effects, such as reduced productivity, particularly when dealing with complex information with limited face-to-face contact (Battistón et al. 2017). During the COVID-19 pandemic, female and low-paid workers working from home reported lower productivity, which was explained, in part, to women being disproportionately burdened with childcare duties (Etheridge et al. 2020). Furthermore, homeworking has been implicated in reduced wellbeing, with some employees highlighting an inability to mentally “switch off” when working from home (Felstead and Henseke 2017), and increased feelings of loneliness, irritability, and stress (Mann and Holdsworth 2003).

Thus, existing literature regarding the benefits and drawbacks of remote working is mixed. Nonetheless, an increased prevalence for homeworking is set to continue (Felstead 2021); therefore, employers should seek to understand how best to support employees to ensure the maintenance of productivity and wellbeing over the long-term. A pertinent issue is the concept of effective time-use when working from home. Specifically, understanding and optimising how employees engage with rest breaks whilst working from home is critical to managing the longevity of employee wellbeing and productivity.

A rest break is defined as an episode of the workday when employees shift attention away from work tasks (Hunter and Wu 2016). According to UK legislation, workers have the right to at least one uninterrupted 20-min rest break per working day, should they work more than 6 h a day (Gov.uk 2021) and this break can be used for various purposes including a scheduled lunch break. Despite this, a large proportion of UK workers do not engage with rest breaks fully: over half fail to take a full lunch break and a third do not leave their workplace once during the day (Totaljobs 2017). Research on this topic frequently focuses on shift workers, finding that rest breaks are beneficial in preventing fatigue-based risks and accidents (Folkard and Lombardi 2006; Folkard and Tucker 2003). Beyond shift work, in a sample of 86 white-collar workers, social and relaxation microbreaks (short voluntary breaks while at work) were associated with reduced negative affect, while cognitive focused microbreaks (e.g. reading short magazine articles, making personal plans, or surfing the Internet) contributed negatively, i.e. aggravated impact of work demands on negative affect (Kim et al. 2017). Additionally, self-initiated short breaks have been associated with increased work engagement in an office environment (Kühnel et al. 2017). Recommendations for working at home during the pandemic included the use of small breaks to reduce stress and tension (Birimoglu Okuyan and Begen 2022), yet the effects of regular rest breaks on productivity and wellbeing were not measured, which could highlight new challenges and opportunities for employers to support their workforce.

With the trend towards working from home set to continue (Felstead 2021) many companies are now adopting hybrid models of employment, blending home and office working (Office for National Statistics 2022). Thus, exploring attitudes towards engagement with rest breaks at home and capturing the number of breaks taken, wellbeing and productivity in a sample of UK white-collar workers could help to inform employers regarding the support needed, in the transition to a new era of working practices. The current research directly addresses this evidence gap, and sets out two specific aims:

1. To explore attitudes and perceptions towards factors that may facilitate or create barriers to taking rest breaks while working at home.
2. To examine rates of, and relationships between, self-reported wellbeing, productivity and rest break behaviour whilst working from home.

## Methods

### Design

We adopted a mixed-methods design to investigate the complex nature of health and productivity outcomes (Dures et al. 2011). Qualitative and quantitative data were used in a complementary (Greene 2008), convergent parallel design. Within an online survey, free-text responses were used to gather qualitative data, while quantitative self-report measures were used to gather rest break behaviour, productivity, wellbeing.

### Participants and procedure

Participants were full-time employees at a global organisation specialising in manufacture and distribution. Participants were required to be UK-based office workers, at least 18 years of age, and working from home at least some of the time. The survey was circulated via internal channels to a select group of UK-based employees. Data were collected between the 6th September and 15th October, 2021. During this time, there were no COVID-19 changes in the UK working practices (Institute for Goverment analysis 2021). Ethical approval was granted by the Manchester Metropolitan University Research Ethics Committee (REF 32303). Two-hundred and fifty participants started the survey, *n* = 153 participants provided complete qualitative responses, and *n* = 140 provided complete quantitative responses, this equated to a 56% completion rate.

### Measures

Self-reported demographic information included age, ethnicity, gender, employment type and role within the organisation.

#### Perceptions and attitudes to taking rest breaks

Participants were asked a series of open-ended questions relating to attitudes and perceptions towards factors that may facilitate or create barriers to taking rest breaks while working at home. For example, “What organisational/personal barriers prevent you from taking effective breaks when working from home?”.

#### Rest break behaviours

Participants self-reported prevalence (number of breaks taken per day), number of times they left their designated workstation per day, and the number of times they took breaks outside per day.

#### Productivity

Productivity of white-collar workers is historically difficult to measure (Schroeder Roger et al. 1985). We used the World Health Organisation Health and Performance Scale (HPQ), specifically the 7-item presenteeism subscale (Kessler et al. 2003). The HPQ is a self-report instrument designed to estimate the workplace costs of health problems related to reduced job performance, sickness absence and work-related accidents and injuries. Seven items have been included in the presenteeism sub scale. Items are measured on a 4-point Likert scale, ranging from 0 “none of the time” to 4 “all of the time”. Items two to seven were reverse scored, after which all items were averaged. Scores ranged from 1 to 7, with higher scores indicating higher levels of productivity (Cronbach’s alpha = 0.66). We have previously used this measure to index productivity in white-collar workers (Braithwaite et al. 2022).

#### Wellbeing

Wellbeing was assessed using the Short Warwick-Edinburgh Mental wellbeing scale which includes 7-items. The scale captures concepts such social relationships, sense of purpose and feelings of happiness. Items were measured on a five-point Likert-type scale, from 1 = ‘none of the time’ to 5 = ‘all of the time’. Scores ranged from 7 to 35, and were transformed following scale guidelines to create a standardised score, in which higher scores equated to higher wellbeing levels (Tennant et al. 2007), (Cronbach’s alpha = 0.81).

### Qualitative analysis

Open-text responses from the survey were analysed using reflexive thematic analysis as outlined by Braun and Clark (Braun and Clarke, 2006). The process of analysis involves six stages. (1) Familiarisation with the data, (2) Code generation, (3) Initial theme generation, (4) reviewing of themes, (5) naming and defining themes, (6) Narrative synthesis of data and integration of literature. Overarching themes were analysed deductively (organisational/personal), while all other themes were developed inductively. Two researchers undertook the analysis (LW and SS). LW read the free-text responses. Codes were added manually to each response, focusing on reoccurring thoughts and ideas within the text. Responses were broadly categorised into positive, negative or neutral. The second coder (SS) then independently checked the coding of responses. Where discrepancies were identified between the two coders, agreement was met through discussion. After the coding process, LW organised codes into initial themes, which was then checked independently by the second coder. Lastly, both coders met to discuss the final order of themes and development of the thematic map. As before, discrepancy between coders was resolved through discussion.

### Statistical analysis

Data checks were used including: Shapiro–Wilk tests, histograms and Q–Q plots for normality. Descriptive statistics were presented as percentages, means and standard deviations. Job roles were grouped based on level of experience to create three role types: Executive or Senior (Executive or senior staff), Professional (professional staff or technician), Support (Customer service, admin or secretarial).

Pearson correlations were calculated to test relationships between productivity, wellbeing, and rest break behaviors. Four stepwise regression models were then constructed. The dependent variable (mental wellbeing or productivity) was entered, the first step of predictors included outside breaks or total breaks taken, the second step was the inclusion of the demographic characteristics of age and gender. Assumptions and tolerances of regression models were checked to control for multicollinearity. All variables entered were continuous in nature, apart from gender which was entered as a dichotomous (Male vs Female). Effect sizes were calculated and reported (r) and a probability of significance considered at *p* < 0.05. Further measures were not entered as covariates in the analysis due to restrictions in sample size.

## Results

### Qualitative results

**Fig. 1:**
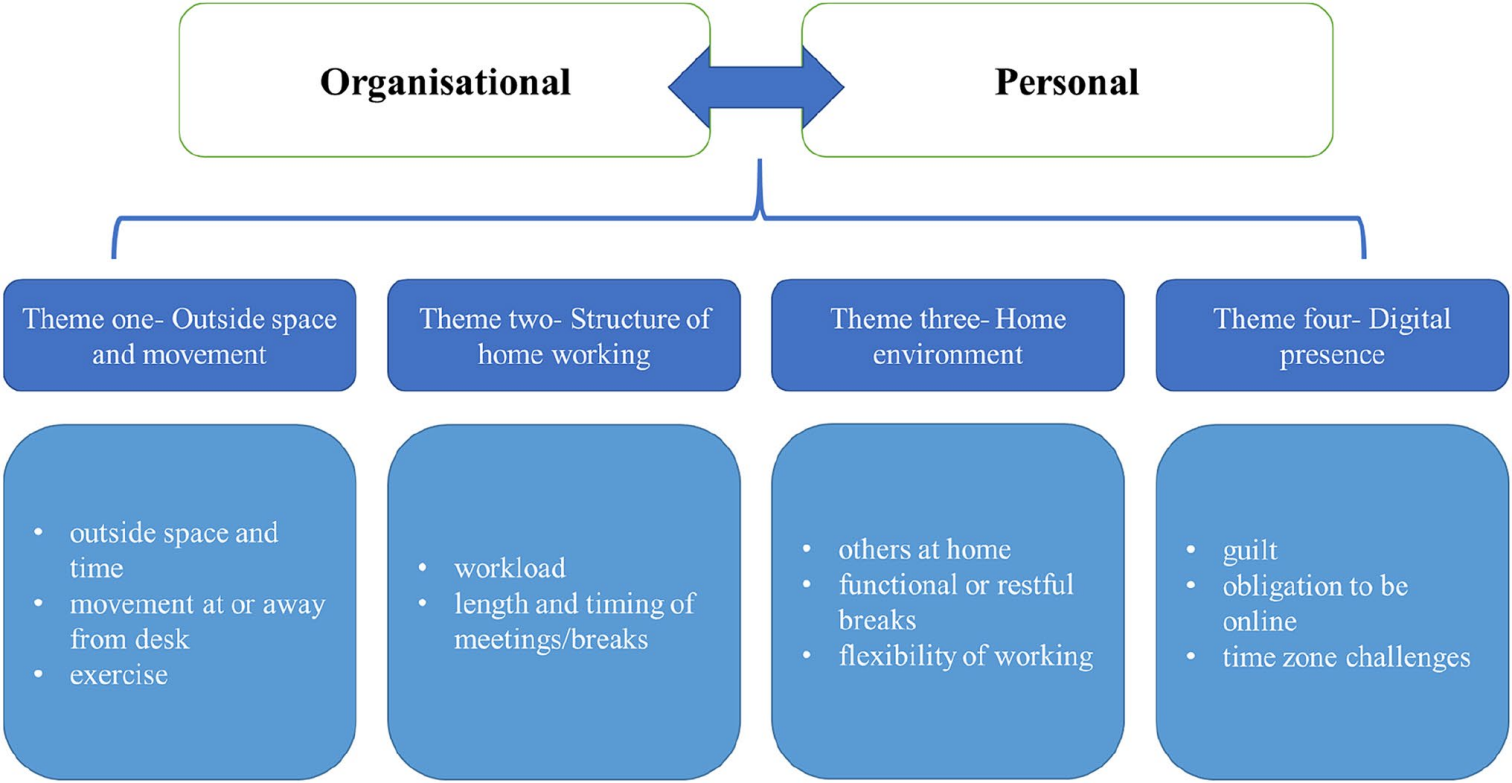
Thematic map. Demonstration of the structure of the themes constructed from the qualitative free-text responses

Two overarching themes: (1) Personal and (2) Organisational took precedence over all other themes identified. Positioned under the overarching themes are four main themes each including a number of subtheme points. The themes include 1. Movement outside, 2. Structure of home working, 3. Home environment, and 4. Digital presence. Figure 1 displays a thematic map, depicting the visual representation of the theme structure.

Below outlines a description of each theme, while Table 1 provides example illustrative quotes.

**Table 1:** Summary table of illustrative quotes

**Theme one: Outside space and movement**
Organizational
*Outside space time:* “I do make the most of lunchtimes to get outside though which i wouldn’t do at work as i would lunch with colleagues instead” “When I was physically going to the office I would meet a friend most lunch times and take a walk outside for at least 30 min—some days now I don’t leave the house!”
*Movement away/at desk:* “It’s a different environment in work as there are different places to take a break, and different people to talk to.” “I can feel ‘desk-bound’ because all my work and meetings are at the desk. It can be difficult to find time for breaks or to go outside. I can go all day without leaving the house.” “At work I would take a 'rest' break between meetings as I would move from my desk and between meeting rooms, or even have a moving meeting. Now when all meetings are done through my laptop I have no need to move from my desk, and often on busy days can have 3 or 4 h at my desk without moving”
*Exercise:* “Normalise flexible working in the office setting so it carries through to home working. For example, if my exercise class of choice is at 9am or 2 pm, why should it be that I cannot go and return again?”
Personal
*Outside space time:* “I try to eat lunch and take short walk outside at lunchtime everyday to get some fresh air and movement.” “I wander round the garden, feed the fish, or look at wildlife and it calms me down and lets me stretch my legs”
*Movement away/at desk:* “The other thing I can do is continue to get some fresh air even if for 10 min as that really helps me reset my mind.” “Take control of my own work load, effective time management and making sure I do move around and get outside when I can.”
*Exercise:* “Luckily I had a dog to look after so I've been doing 3 walks a day, one before work, one during a lunch hour (followed by making lunch), then one after work. I often go get up and leave my desk and do some stretches” “Always make an effort to do some form of exercise outside such as walking, running, cycling. This is certainly easier when working from home—can change and shower more easily.” “Try to do my exercise first thing in the morning, then it's done and I can’t put it off later! Possibly take small breaks of 10–15 min rather than looking for 60–90 min to do my 10,000 steps in one go, but it’s more enjoyable to do one bigger walk. Maybe a mixture. Possibly try to meet someone else or have an objective of the exercise. I do try to use my Fitbit to kick me into action to make me take at least 250 steps per hour—it's a lot better than nothing. I should set some exercise objective online like ‘climbing Everest on the staircase’ or ‘walking the Pennine Way’ or something,”
**Theme two: Structure of home working**
Organizational
*workload*: “workload expectations from management Agendas are simply incredibly full and we work across all time zones so there isn't a clear lunch break for everyone.” “logistics problems, material shortages and incidents have increased the normal work load and involve needing to talk to and work with others—which is all now tied to a computer”
*Length/timing of meetings:* “Meetings running over or due to different time zones being early/late, bad connections, find it difficult to switch off at end of the day.” “Back-to-back meetings. The need to be on camera makes it more difficult to sneak off to get a cup of tea during a meeting, but I sometimes do if I’m just listening not speaking. There is no natural start or finish time delineated by travel to/from office so I carry on until I'm finished or run out of steam, unless I arrange other outside appts. Due to “flexible working” and “global working” there are always people trying to contact me.” “Make it the "done thing" to set meetings to finish at 10 min to the hour, so even if you have a full agenda you have built-in gaps in between where you can take a break.” “institutionalise shorter and more effective meetings” “Scheduling meetings should occur during core hours: 9.00–12, 2–5. However meetings shouldn’t be back 2 back as it is but only for 45 min. MAX and this then give 15 min per hour to walk and move”
Personal
*Workload*: “Thinking I need to finish/ complete things” “Personal pressure to achieve/deliver” “Desire to complete work on the ever-increasing "To Do" list” “working with multiple time zones, high workload”
*Length/timing of meetings*: “Block out time and make myself go out. Decline meetings that happen over lunch times.” “plan time better. block time in diary to take breaks. get outside and walk—stretch to ensure take good amount of breaks.” “Set boundaries, set specific break times and stick to them—making the team aware that I will be away from my desk then, so to contact me outside of those times.” “Perhaps be more assertive in leaving meetings so that there's a break Continue to check work priorities with my Line Manager; the ever-increasing To Do List also impacts upon potential breaks when working on site and is not limited to working from home”
**Theme three: Work environment**
Organizational
*Others in working space*: “Lack of regular interaction with my team makes me feel isolated at home. Too many 'broadcasting' meetings (where I just have to listen to others).” “I'm normally on my own so I don't get to interact with my colleagues but it's also very peaceful to be able to spend time in the garden.”
*Functional versus restful breaks*: “Usually they are very short and limited for a specific purpose, i.e. go to the toilet or make a drink. If I were at the office, I'd have longer breaks as I'd have informal chats with colleagues when doing so, as well as when moving from one side of the building to the other I'd move/rest more.”
*Flexibility of working*: “Normalise flexible working in the office setting so it carries through to home working. For example, if my exercise class of choice is at 9am or 2 pm, why should it be that I cannot go and return again?” “Stop micromanaging, develop a good level of trust, and introduce flexible working times. Not to expect a normal working hour day due to external distractions.” “Our organisation has been great re allowing us to work flexible hours. We work internationally so can have meetings at very different times so have a degree of fluidity when it comes to managing working hours. Maintaining this would be great. Letting people know what is and isn't reasonable in terms of acceptable break times.”
Personal
*Others in working space*: “I also don't want to disturb other family members by taking a break, as they may be in the middle of a meeting—I have no way of telling until I leave my office.” “others in house draw me into problems and conversation wasting time and energy”
*Functional versus restful breaks*: “They generally feel less like a break as I often end up using them to do a small housework task instead I do make the most of lunchtimes to get outside though which i wouldn't do at work as i would lunch with colleagues instead” “often quickly to grab a tea or coffee from downstairs. Find these are not very restful as I will multi-task, e.g. while the kettle boils I may put a wash on, empty the dishwasher.”
*Flexibility of working:* “Block out more time in my diary but this is difficult because I try to be flexible (and people clearly can't book meetings if I block out time). I try to pre-book my lunch break as often as I can.”
**Theme four: Digital presence**
Organizational
*Guilt:* “Sometimes I worry about leaving my laptop and it looking like I'm not working” “maybe as teams we need to decide on some set break times so you can keep yourself in a routine and not feel guilty about the need to be ever present.” “ensure that it is clear from higher management that breaks are normal and encouraged”
*Obligation to be online*: “Sometimes feel with Team it displays if you're 'away' and how long you have been away for. I am more conscious of the length of my breaks.” “its hard to take breaks as feel like always need to be present online when at home” “Ensure that all Line Managers are taking regular breaks and that they are fostering a culture with their team that it is ok to take regular breaks and that for everyone's wellbeing it is essential”
*Time zone challenges:* “Number of meetings and being a global business there is a need to adapt to different times.” “working with Europe and the time difference can mean that UK break times are interrupted.” “Agendas are simply incredibly full and we work across all time zones so there isn't a clear lunch break for everyone.” “Allotted time where no meetings or calls are allowed—wellbeing Wednesdays were good but doesn't work for global teams.” “For me the key thing is that whatever is implemented needs to be almost universal in a way (could be the principle and not the exact time, per se) because otherwise people like us who work with multiple countries cannot really do much.”
Personal
*Guilt:* “Find it much harder to bring myself to take breaks, hit with a sense of dread and guilt in case it reflects badly on my performance.” “(need to) Feel less guilt about going for walks and enjoy spending breaks on my own” “feeling guilty for taking time away from desk” “personally I want to make sure that i get as much done (or more than before at work). i don’t have much work that i can do away from my desk/ laptop and so feel guilty 'taking time out' even thought I know in theory it should boost my productivity later.” “To set time in our calendars, so we don’t feel guilty of taking breaks working from home” “Ensure you have time planned in the diary for breaks and not accept meetings all day back to back with no breaks. Don't feel guilty for having 5 min in the garden.”
*obligation to be online:* “its hard to take breaks as feel like always need to be present online when at home” “Not giving myself permission to be kind to myself and the thought that I am somehow not doing full hours if I go out of the house during the day.” “block time out for breaks make sure I go into another room and step away from the computer not bring my coffee up to my desk to drink, but have it downstairs away from my desk encourage team members to take breaks do not instant message someone when you can see they are away from their computer but wait until they are back (eg away on teams messenger ap)” “silence my computer when I’m on a break so I don’t feel I have to immediately respond to teams and emails”
*Time zone challenges:* “Constant call requests, different time zones makes it hard to prioritise break if it’s the only time someone can do”

### Theme one: movement and outside

This theme encapsulates how lack of movement and lack of outside space has a negative impact on rest break behaviours, while an increase in these factors could increase productivity. Three subthemes make up the theme, including (i) outside space and time, (ii) movement at or away from desk, and (iii) use of or lack of exercise.

In the first sub-theme, *outside space and time,* from an organizational perspective participants explained how prior to working from home they may not have taken advantage of outside space due to the office environment. However, many participants stated that when working from home they personally made an effort to take short walks outside. This is in contrast to the second subtheme *movement at or away from desk.* From an organisational view, the office environment is described as more conducive to taking breaks, with more areas for breaks and a variety of opportunities to leave the desk, whereas at home participants felt ‘desk-bound’ as all interactions, be it meetings or social, are facilitated through the screen. However, participants also took personal responsibility to make sure they incorporated breaks away from the desk. Lastly, the subtheme *exercise,* through an organisational lens, participants felt more could be done to promote exercise behaviors, mostly via promoting acceptability of flexible working habits such as being able to take longer breaks in the day to attend exercise classes. At home, participants took personal ownership of their exercise behavior, fitting it in around working activities and monitoring this objective. This theme demonstrates how movement and time spent outside is an integral part of the experience of rest breaks when working from home.

### Theme two: structure of home working

This theme describes the patterns and restrictions of home working that contribute to shorter and fewer breaks, leaving employees feeling over worked. Subthemes include (i) workload and (ii) length and timing of meetings.

Within the first subtheme *workload,* from an organisation perspective, participants expressed being overwhelmed by the pressure and amount of work set by the organisation which create time barriers to employees taking breaks. Personally, participants also cited putting pressure on themselves to complete tasks, which detracted from taking breaks. The second subtheme, *length and timing of meeting,* adds weight to this organisational pressure, as participants report an increased number ‘back-to-back’ meetings online. Participants explain how creating expectations around gaps between meetings would allow individuals to take breaks, increase movement and create less fatigue, overall increasing productivity as a result. Some participants personally felt responsible for planning their own breaks between meetings or declining meetings over lunch to facilitate breaks, rather than expecting organisational-wide changes to occur. The theme shows how when working from home, different challenges occur in terms of managing workload and organization of meetings, all of which impact attitudes towards, and barriers in, taking breaks.

### Theme three: home environment

The third theme describes elements of home working, in contrast to previously experienced office environment, that impact rest breaks taken. Three subthemes can be used to describe this, including (i) others in working space, (ii) functional versus restful breaks and (iii) flexibility of working.

The first subtheme *others in working space,* from an organisation perspective, describes how the loss of regular interaction with team members leads to fewer breaks being taken and feelings of isolation. Alternatively, participants also cite that reduced interaction enables them to spend breaks outside when working from home. However, when working from home *others in working space* became a barrier for some participants as they did not wish to disturb other household members when taking breaks, or they found that others in the household were distracting from formal break behaviour. Participants also indicated that prior organisational structure enabled restful breaks within the subtheme *functional verses restful breaks* as participants describe how an office setting facilitated taking longer breaks, becoming ritualised within the social environment including informal chat with colleagues and moving around the building. In the home environment, participants are drawn into completing housework and tasks that would not usually accompany the working day. This led to breaks being very functional in nature rather than be restful. Lastly *flexibility of working* captures how participants felt the organisation should normalise flexible working practises to alleviate the pressures of the home environment, this includes trusting employees to personally manage their own time while setting boundaries in terms of what is reasonable and acceptable in taking breaks.

The theme identifies how the loss of the office environment and face-to-face contact with colleagues, is replaced by challenges in the home environment which create significant barriers to taking restful breaks.

### Theme four: digital presence

The final theme, *Digital presence*, describes the unique situation of working from home with the heavy reliance on technology to facilitate all aspects of work. The theme is made up of three subthemes *guilt*, *obligation to be online* and *time zone challenges*.

From an organisational stance, individuals felt *guilt* was imposed from the online structure of work, linking to workload pressures, as participants felt guilt when taking breaks due to the fear of not being considered working hard enough. From a personal view, *guilt* was also a significant deterrence to breaks, yet this was communicated as internalised guilt surrounding the impact of breaks on performance rather than views of others. However, participants did recognise that this ‘guilt’ was not a rational thought, and that taking breaks would in fact be likely to increase productivity. Guilt has a close relationship with the second subtheme *obligation to be online,* as participants noted the technical feature of video conferencing and messaging software that informs team members and colleagues of online or inactive status, created pressure and obligation to appear as active online, in turn preventing breaks being taken away from the desk. One-way participants noted the organisation could manage this is through team leaders and managers setting examples for online working habits and breaks. Lastly *time zone challenges* were noted. Due to the international nature of the workplace, employees have to adapt their working schedule to different time zones; however, with this comes issues as many participants stated there was not a clear period to step away from the desk. The individual feels the effects of this pressure, as participants struggled to plan and manage breaks when constant call requests were received from different time zones. Participants indicated that changes around break behavior could be communicated to international partners to impact positively on break behaviour.

This theme encapsulates some of the unique challenges faced during when working from home, organisations can reflect on how to effectively deliver messages regarding the need to be online and taking breaks, whereas the individual can work on prioritising breaks over online presence.

## Quantitative findings

### Prevalence of rest breaks, wellbeing and productivity

Table 2 outlines participant demographics and self-report levels of rest breaks, wellbeing and productivity. On average, participants reported taking 3.69 breaks per day (*N* = 140), of which an average of 2.66 were taken away from their designated workspace, and just 1.20 were taken outside. The mean rating for productivity was 3.90, the mean wellbeing score was 21.06.

**Table 2:** Participant demographic information

	**%**	Mean (SD)	Range
Age (years)		42.28 (.90)	19–64
Gender			
Female	82.9		
Male	17.1		
Ethnicity			
White British	79.3		
White Irish	.7		
White Other	14.3		
Mixed/multiple ethnic groups	.7		
Asian/British—Indian	3.6		
Asian/Asian British—Pakistani	.7		
Any other ethnic group	.7		
Employment type			
Permanent	87.9		
Fixed Term	8.6		
Contractor	3.6		
Role type			
Executive or Senior	26.7		
Professional	50.4		
Support	22.9		
Number of days working from home per week		4.29 (.90)	2–6
Number of working hours in a day		8.74 (1.92)	7–24
Number of breaks typically taken per day		3.68 (1.33)	
0	.7		
1	22.9		
2	22.1		
3	30.0		
4	10.7		
5+	13.6		
Number of times left designated work area to take a break		2.66 (1.45)	
0	0		
1	31.4		
2	16.4		
3	22.9		
4	12.9		
5+	16.4		
Number of times going outside to take a break		1.20 (.54)	
0	0		
1	85.7		
2	9.3		
3	4.3		
4	.7		
5+	0		
Productivity		3.92 (.46)	2.57–5.00
Wellbeing		21.09 (3.15)	9.51–35.00

### Correlations

The correlation matrix presented in Table 3 indicates that the three measures of rest break activity were related, i.e. those people who reported more rest breaks overall also reported more rest breaks away from their desk and outside. Additionally, wellbeing and productivity were positively correlated, and the number of breaks taken outside was positively associated with increased wellbeing.

**Table 3:** Correlations between rest breaks taken, productivity and wellbeing

Measure	1	2	3	4	5
1. Number of rest breaks	1				
2. Number away from desk	.68**	1			
3. Number outside breaks	.31**	.43**	1		
4. Productivity	−.10	−.03	−.06	1	
5. Wellbeing	.08	.11	.17*	.31**	1

*p < .05, **p < *.001*

### Regression analyses

See Table 4 for summary of regression models 1 and 2. Step one of Model 1 indicates a significant association between self-reported outside rest breaks and wellbeing, which accounts for 3% of the observed variation in wellbeing. However, when covariates are entered in step 2 the association becomes non-significant, though the effect sizes remain similar (*B* = 1.02 and *B* = 0.98, respectively). No significant model or contribution of individual predictors is observed for Model 2, including productivity. The impact of total breaks taken on wellbeing and productivity analysis is presented in Table 5. Models 3 and 4 display the lack of influence of rest breaks taken on both wellbeing and productivity.

**Table 4:** Association between taking rest breaks outside and self-reported wellbeing and productivity, Stepwise regression model coefficients

Model	*t*	*p*	*B*	B 95% C LL UL	β	F	*p*	*R*^2^
*Model 1- Wellbeing*								
Step 1						4.06	.046	.030
Outside breaks	2.01	.046	.99	.02, 1.99	.17			
Step 2						1.40	.246	.030
Outside breaks	1.91	.059	.95	−.04, 1.94	.16			
Age	.18	.853	.02	−.05, .06	.016			
Gender	−.40	.691	−.28	−1.68, 1.29	1.12			
*Model 2- Productivity*								
Step 1						.55	.460	.004
Outside breaks	−.74	.460	−.05	−.20, .09	−.06			
Step 2						.225	.879	.005
Outside breaks	−.77	.440	−.06	−.20, .09	−.07			
Age	.36	.717	.00	-.01, .01	.03			
Gender	.03	.976	.00	−.20, .21	.00			

Model 1 df = 1, 139/ 3, 139, Model 2 df = 1, 139/ 3, 139.* N* = 140

**Table 5:** Association between number of breaks taken and self-reported wellbeing and productivity, Stepwise regression model coefficients

Model	*t*	*p*	*B*	B 95% C LL UL	β	F	*p*	*R*^2^
*Model 1- Wellbeing*						1.65	.201	.010
Step 1								
Number of breaks	1.29	.201	.24	−.13, .60	.11			
Step 2						.686	.562	.020
Outside breaks	1.23	.223	.23	−.14, .59	.11			
Age	.41	.683	.01	−.04, .06	.04			
Gender	−.51	.614	−.36	−1.76, 1.05	−.04			
*Model 2- Productivity*						.10	.752	.001
Step1								
Number of breaks	−.32	.752	−.01	−.06, 05	−.03			
Step 2						.06	.981	.001
Number of breaks	−.32	.750	−.01	−.06, .05	−.03			
Age	.27	.789	.00	−.01, .01	.02			
Gender	.09	.931	.01	−.20, .22	.01			

Model 1 df = 1, 139/ 3, 139, Model 2 df = 1, 139/ 3, 139.* N* = 140

## Discussion

Taking effective breaks in the workplace has been associated with benefits to wellbeing and productivity in several roles, including shift and office workers (Folkard and Lombardi 2006; Folkard and Tucker 2003; Kim et al. 2017; Kühnel et al. 2017). However, the prevalence of taking breaks and factors impacting on taking these breaks is not widely understood in the context of home working. The current research sought to understand the attitudes and perceptions of the facilitators and barriers impacting taking rest breaks while working from home and investigate the influence of breaks taken on levels of wellbeing, and productivity in a sample of UK-based employees working from home.

In the current study, qualitative findings provide rich insight into the perceived barriers and facilitators to rest break behaviour, adding understanding beyond what quantitative measures can provide. Two overarching themes to the analysis were personal and organisational. Four further themes were outlined these included Movement outside, Structure of home working, Home environment and lastly Digital presence. Quantitative findings indicate a weak association between the number of breaks taken outside and increased wellbeing scores, whereas no influence of total number of breaks taken was observed.

Both qualitative and quantitative data suggests that increasing outside breaks could positively impact wellbeing. Specifically, to the qualitative themes illustrate this point as taking outside breaks was perceived to increase how restful the breaks were, in turn increasing productivity. Prior research has extensively investigated the benefits of being in nature for overall wellbeing and stress reduction (Gilbert 2016; Reyes-Riveros et al. 2021), while during the pandemic, sedentary behavior associated with lockdown and home working were associated with increased stress and impaired mental wellbeing (Savage et al. 2020). An important aspect to outside breaks identified by participants was exercise. Higher levels of physical activity have been suggested to increase overall wellbeing and productivity in the work place, yet increased sitting time had a negative impact despite levels of activity (Puig-Ribera et al. 2015) which could be ameliorated through increased movement (Foley et al. 2016). Therefore, the importance of reducing sitting time and increasing outside breaks is supported, with the additional benefit of including physical activity into these breaks.

Surprisingly, no other significant effects were observed when looking at the quantitative findings, yet in depth analysis provided from the qualitative responses help to illuminate why this may be the case. First, unique to the qualitative themes developed, the difference in structure of work between the office and home is apparent. Participants expressed how workloads and back-to-back meetings were perceived to be increased, reducing the time available to take breaks. Qualitative findings reported during the pandemic mirrored this theme (Karl et al. 2021), indicating that individuals experienced ‘bad’ meetings (characterised as being overly long and running over), which was linked to reduced wellbeing (Luong and Rogelberg 2005; Rogelberg et al. 2006). Quantitative findings may, therefore, be skewed during this time, as the number of breaks available to take is drastically reduced. Another prominent experience of individuals working from home was the potential for work–home interference. Participants felt the home environment was not suited for productive work and often rest breaks would become an opportunity to complete functional tasks in the home. Working from home is proposed to cause a range of distractions that influence stress levels and exhaustion in staff (Bergefurt et al. 2021). Participants cited using their own rest break behaviors to engage in at-home tasks, as such when wanting to quantify breaks taken, individuals may have therefore report taking a break, when in reality the break was functional in nature, reducing the impact this break may have had on both wellbeing and productivity. The current qualitative findings highlight the contextual details of working from home, often missed within a survey design.

The self-regulation theory (Hall and Fong 2007) can be used to explain the interference of breaks when working from home. The theory emphasises the individual’s own ability to guide their own activities by setting standards. Work breaks are taken as an autonomous action, yet traditionally they take place within an organisational setting (the work place) that sets social and situational demands (Heatherton and Baumeister 1996). Within the workplace, break behavior is self-regulated through situational demands of the office, whereas when working from home these conventions are removed, leading to potentially less self-regulation of rest break behaviour, in turn increasing interference in break behaviours through demands of the home environment. Qualitative findings also highlighted the digital presence associated with working from home, linked to the structure of home working is. Personally, individuals felt guilty when they did not appear as ‘online’, which led to reduced rest break behaviour. Whereas others recognised that the organisation should set an example through actively taking time away from the desk. Time zone differences often also disrupted this. As with taking outside breaks and exercise behaviour, individuals felt the management of meetings and management of the online presence both their personal responsibility (e.g. putting breaks in the day and over lunch), and the organisation had a duty to set a precedent for ending meetings sooner and being away from the desk to allow breaks to occur. For example, authentic leading styles have been shown to increase productivity levels of staff (Daraba et al. 2021), furthermore when authentic leaders demonstrate behaviors such as setting effective work-life boundaries, individual employees are more likely to engage in this behaviour (Braun and Peus 2018). The Theory of Planned Behaviour gives weight to the role of authentic leaders (Ajzen 1991), which indicates that change in behaviour will occur if the individual (i) perceives they have control over their own behaviour, (ii) the behavior is a subjective norm and (iii) there is a positive attitude associated with the behaviour (Blasche et al. 2021). Individuals working from home may feel like they do not have control over taking a break due to high workload demands, furthermore they may perceive that the norm is to not take many breaks and to be seen as ‘online’ during the working day. Lastly, individuals may think that taking a break reflects badly on their performance. Authentic leadership could be utilised to demonstrate the positive of taking breaks, and create company-wide ‘social norms’ around being away from the desk and not ‘online’ for all hours of the day.

## Limitations

The study sample included employees from one global organisation; therefore, results may not be generalisable. Furthermore, the current data were collected when COVID-19 restrictions for working were put in place in the UK, meaning a high amount of home working activity was the precedent across a range of sectors. Although restrictions no longer apply, hybrid working (a combination of at home and on site working) is still a popular choice for many individuals and companies (Office for National Statistics 2022). Finally, as indicated above quantitative methods may not have captured the rich contextual details associated with taking breaks when working from home, despite this the qualitative data within this study adds great depth to our understanding.

## Conclusions and implications for the workplace

This study highlights several implications for employers supporting workers at home, through providing rich insights obtained from qualitative findings, going beyond what could be achieved through solely fixed response survey design. First, employers should strive to promote movement and exercise throughout the working day, specifically in outside space, which could be emphasised through authentic leadership and setting company-wide ‘social norms’. Second, employers could seek to promote self-regulation of rest break behaviours in the home environment, providing training or resources to help prevent work–life interference and in turn increasing productivity. Furthermore, when attempting to measure behaviour centred around rest breaks when working at home, researchers and employers should be aware of the differences in types of breaks taken, which may heavily influence research findings.

## Data Availability

Restrictions apply to the availability of data, due to the sample and information gathered. The data are, however, available from the authors upon reasonable request.
